# Coxsackievirus A24 causing acute conjunctivitis in a 2023 outbreak in Vietnam

**DOI:** 10.1016/j.ijid.2024.107133

**Published:** 2024-06-13

**Authors:** Huy Tran, Thao Ha, Linh Hoang, Yen Tran, Kevin Ruder, Lina Zhong, Cindi Chen, YuHeng Liu, Danny Yu, Thomas Abraham, Armin Hinterwirth, Michael Deiner, Travis C. Porco, Thomas M. Lietman, Thuy Doan, Gerami D. Seitzman

**Affiliations:** 1 Hai Yen Vision Institute, Ho Chi Minh City, Vietnam; 2 Francis I. Proctor Foundation, University of California, San Francisco, USA; 3 Department of Ophthalmology, University of California, San Francisco, USA

**Keywords:** Coxsackievirus, Epidemic, Conjunctivitis, Keratoconjunctivitis (EKC), Vietnam

## Abstract

**Objectives:**

To determine the associated pathogen during the 2023 conjunctivitis outbreak in Vietnam

**Methods:**

RNA-sequencing was used to identify pathogens before and during the outbreak.

**Results:**

24 patients with infectious conjunctivitis between March and October 2023 from Hai Yen Vision Institute in Vietnam were swabbed. Coxsackievirus A24v was the most common pathogen identified. Phylogenetic analysis of these strains demonstrates similarities to the Coxsackievirus identified in the 2022 India outbreak. Human adenovirus D was also circulating. Ocular findings of tearing, purulence, and itching were common in this outbreak.

**Conclusions:**

Multiple viruses can co-circulate during conjunctivitis outbreaks. Hemorrhagic conjunctivitis, commonly associated with coxsackievirus conjunctivitis, was not a common clinical sign in this outbreak. Repeat genetic surveillance, with the notable inclusion of RNA virus detection strategies, is important for outbreak detection.

## Introduction

During September and October of 2023, infectious conjunctivitis case numbers soared through regions of Asia including Vietnam [1]. The causes of conjunctivitis epidemics in Vietnam are not typically investigated, as is true worldwide. Adenovirus, commonly assumed, but rarely confirmed is one cause of conjunctivitis in Vietnam [2]. This study aims to determine the pathogen(s) responsible for this outbreak and use phylogenetic analysis to characterize a potential path of infection. The Hai Yen Vision Institute (HYVI) in Ho Chi Minh City, Vietnam, is one participating center in a prospective international infectious conjunctivitis study called SCORPIO (Seasonal Conjunctivitis Outbreak Reporting for Prevention and Improved Outcomes). In this study, RNA-sequence analysis (RNA-seq) was used to investigate pathogens associated with infectious conjunctivitis. Swabs were collected before and during this epidemic.

## Materials and methods

This research was approved by the Institutional Review Boards of both the HYVI in Vietnam and the University of California San Francisco (UCSF) in the United States. This study adhered to the tenets of the Declaration of Helsinki. Samples were obtained from March through October 2023 at the HYVI. Inclusion criteria required signs and symptoms suggestive of acute infectious conjunctivitis for less than 14 days. Patients with allergic or medicamentosa conjunctivitis are excluded. Sample handling, library preparation, sequencing, and bioinformatic algorithms for pathogen identification have been previously described [3]. Briefly, extracted RNA was converted to cDNA. Sequencing libraries were prepared using the NEBNext ULTRA II RNA Library Prep Kit for Illumina (New England Biolabs, Ipswich, MA), pooled, and sequenced on the NovaSeq system (NovaSeq 6000, Illumina, San Diego, CA). For pathogen identification, human reads were removed and remaining non-host reads were aligned to the entire NCBI reference database. Phylogenetic analysis was performed by first aligning the matched reads against the D90457.1 coxsackievirus A24 reference genome using bowtie2 [bowtie2]. “Samtools” and “Bcftools mpileup” commands were then used to build the consensus sequence for regions with a read depth greater than 5 [samtools+bcftools]. Consensus sequences with a genome coverage >80% were then compared to various published enterovirus references to create a time tree by running the “augur” toolkit [augur] [4].

## Results

**Figure 1.**
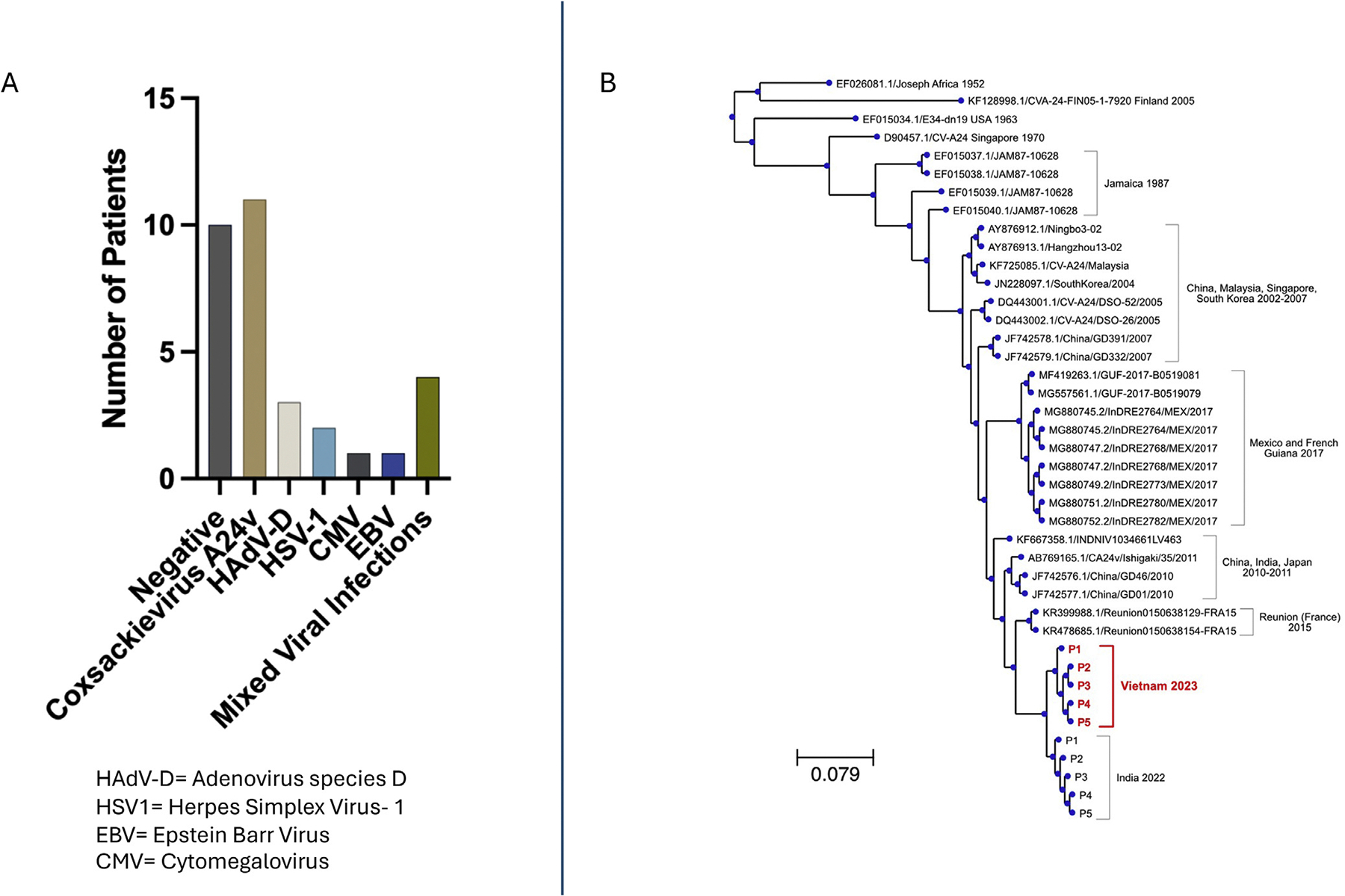
(a) Pathogens identified by RNA-sequence analysis during the 2023 conjunctivitis outbreak in Vietnam (n=24). **(b)** Maximum-likelihood phylogenetic tree based on the genome sequences of CV-A24v isolated from 5 patients from the outbreak in Ho Chi Minh City in 2023 and prior outbreaks, including a prior outbreak in India in 2022 also captured by our study.^4^ The coverage of the genomes for this outbreak ranged from 82% to 91%.

Between March and October 2023, 24 patients were enrolled (Table 1). The mean and median age was 29 years (Standard Deviation [SD] ±11 years, interquartile range 19–36). Patients presented shortly after symptom onset with mean days until presentation of 2 days (SD ±1.5 days). 67% of the patients were female. 38% (95% confidence interval [CI]: 21–57%) had exposure to similarly affected close contacts. Both eyes were affected in 75% (95% CI: 50–80%). Runny nose was the most common systemic co-morbid symptom occurring in 21% (95% CI: 9–41%). Regarding eye symptoms, 92% (95% CI: 73–99%) presented with tearing, 88% (95% CI: 68–96%) with purulent discharge, and 67% (95% CI: 47–82%) with itching. Only one patient presented with conjunctival hemorrhage. No patients had conjunctival membranes, pseudomembranous, or corneal subepithelial infiltrates. RNA-seq identified a contributing pathogen in 14/24 (58%) of the patients (Figure 1 a). Of the positive cases, coxsackievirus A24 (CV-A24v) was identified in 11/14 (79%) and HAdV-D in 3/14 (21%). Human simplex virus 1 (HSV-1) was identified in 2/14 (14%). Epstein Barr Virus (EBV) and cytomegalovirus (CMV) were each identified in one patient. Two patients were co-infected with CV-A24v and HAdV-D. One patient was co-infected with CV-A24v and Epstein Barr Virus (EBV) and one patient with CV-A24v and CMV.

In the samples collected prior to September 2023, 1/9 (11%) were positive for CV-A24v. If collected between September and October 2023, the months Ho Chi Minh City Health Department documented a record high number of conjunctivitis cases, 10/15 (64%) were positive for CV-A24v. Patients presenting to the HYVI in Ho Chi Minh City during this time were more likely to be infected with CV-A24v than patients presenting earlier in the year (*P* < 0.01). Focusing only on the CV-A24v cases, purulence and tearing were both reported in 10/11 (90%) and itching in 7/11 (64%). Phylogenetic analysis from the CV-A24v strains isolated from the patients during this outbreak in Vietnam revealed that these strains were closely related to the CV-A24v strains previously identified in the South India outbreak in November of 2022 (Figure 1 b) [5]. The strains associated with the India and southern Vietnam outbreaks shared a common ancestor previously noted to be responsible for the outbreak in Réunion Island in 2015.

## Discussion

During the autumn of 2023, record cases of infectious conjunctivitis were also documented in India, Nepal, Pakistan, and Vietnam. The results of this study suggest that CV-A24v was likely the etiology of this outbreak. HAdV-D was also identified in Vietnam, indicating that this virus is likely endemic and numerous viruses can co-circulate, even during an outbreak.

Coxsackievirus is a single-stranded RNA virus that can lead to a wide variety of systemic febrile illnesses [6]. The A24 variant, specifically, is commonly associated with acute hemorrhagic conjunctivitis (AHC) clinically [7]. Curiously, in this reported outbreak, only one of the patients demonstrated mild subconjunctival hemorrhage, and zero patients demonstrated pseudomembranes. This indicates coxsackievirus conjunctivitis outbreaks can occur without frank AHC as a clinical sign.

Classic teaching regarding clinical differentiation between pathogens responsible for conjunctivitis suggests that tearing is more common with viral pathogens, purulence with bacteria, and itching with non-allergic conjunctivitis [8]. Here, tearing and purulence were commonly reported symptoms (92% and 88%, respectively), and both were equally prevalent (90%) in the CV-A24v infected patients. Itching may also occur more commonly than previously thought during infectious conjunctivitis with 67% experiencing itching in this study. Itching was also frequently reported in patients with SARS-CoV-2-associated conjunctivitis [9]. All members of the Enterovirus genus, coxsackieviruses included, are known to demonstrate significant genetic diversity, with a high mutation rate contributing to this finding. It is unclear how or if different viral strains, distinguished by genetically distinct lineages, contribute to phenotypic variability of clinical presentation [10]. In addition to genetic diversity, biodiversity over time can be evaluated by phylogenic analysis of pathogens. Phylogenetic analysis of viruses responsible for outbreaks and epidemics can be informative as this allows for comparative genetic relations of varying strains over time. In this study, phylogenetic analysis of the CV-A24v associated with infectious conjunctivitis in Vietnam during September/October 2023 demonstrates that these strains cluster closely with the CV-A24v strains identified in the 2022 India outbreak. Both India and Vietnam’s CV-A24v strains appear to be more genetically related to the CV-A24v isolated from the conjunctivitis outbreak in Réunion Island in 2015 than the strains isolated from the Mexico and French Guinea outbreaks in 2017 [11]. Tracking the spread of viruses between populations can shed light on spatial transmission patterns, help us understand how outbreaks spread, and may improve predictions of future outbreak patterns.

This study is limited by sample size. The clinical course was not studied in this surveillance study. Additionally, 42% of the samples analyzed were negative for a pathogen as determined by RNA-seq. As is common with conjunctivitis, in-office microbiologic testing was not performed. Samples negative by RNA-seq may represent previously treated disease, non-infectious etiologies, cases where the host immune system had already cleared the pathogens, or false negative results attributable to low biomass availability inherent to all ocular sampling.

In this era of emerging and re-emerging infectious diseases, disease surveillance is important. The inclusion of RNA virus diagnostics is critical to unbiased disease surveillance efforts. Clinical signs and symptoms may not be reliable predictors of pathogen types. Additionally, we demonstrate that several viruses may co-circulate during conjunctivitis outbreaks. Lastly, phylogenic comparison of viruses responsible for outbreaks may improve our understanding of spread to better predict future outbreaks.

## Figures and Tables

**Table 1:** Demographics of patients with infectious conjunctivitis between March and October 2023 in Ho Chi Minh City, Vietnam.

		Total	%	(SD)

**Sex**	Female	16	67%	
	Male	8	33%	
**Age**	Years	29		± 11
**Duration of symptoms**	Days	2		± 1.5 **(95% CI)**
**Ocular symptoms**	Tearing	22/24	92%	(73, 99)
	Purulent discharge	21/24	88%	(68,96)
	Itching	16/24	67%	(47,82)
**Exam findings**	Bilateral	18/24	75%	(0,12)
	Sub-epithelial infiltrates	0/24	0%	(0,12)
	Membranes	0/24	0%	(0,12)
	Subconjunctival hemorrhage	1/24	4%	(0,22)
	Pre-auricular lymphadenopathy	1/24	4%	(0,22)
**Systemic findings**	Sore throat	4/24	17%	(6,36)
	Runny nose	5/24	21%	(9,41)
	Cough	3/24	13%	(3, 32)
	Diarrhea	1/25	4%	(0,22)
**Contacts similarly affected**	Yes	9/24	38%	(21,57)

SD=Standard Deviation.

95% CI=95% Confidence Interval, Adjusted Wald Method.

## Appendix. Seasonal Conjunctivitis Outbreak Reporting for Prevention and Improved Outcomes (SCORPIO) Study Group:

*Aravind Eye Hospital, Madurai, India* – Lalitha Prajna, N. Venkatesh Prajna, Ramesh Gunasekaran, Sankalp Singh Sharma, Vishnu Teja; *B.P Koirala Lions Center for Ophthalmic Studies, Kathmandu, Nepal –* Meenu Chaudhary, Sanjeeta Sitaula; *Centre de Recherche en Sante de Nouna, Nouna, Burkina Faso* – Ali Sié, Boubacar Coulibaly, Mamadou Bountogo; *Chulalongkorn University, Bangkok, Thailand* – Thanapong Somkijrungroj, Vannarut Satitpitakul; *Hai Yen Vision Institute, Ho Chi Minh City, Vietnam* – Huy Tran, Linh Hoàng Mai, Thảo Ha. Xuân, Yen Tran; *Hospital Clinico Universidad de Chile, Santiago, Chile* – Cristhian A. Urzua, Fabian Vega, Felipe Salgado, Loreto Cuitino; *Instituto Mexicano de Oftalmología, Santiago de Querétaro, Mexico* – Fernando Pérez Pérez, Jaime Macías Martínez, Van Charles Lansingh; *Khon Kaen University, Khon Kaen, Thailand* – Sukhumal Thanapaisal, Wipada Laovirojjanakul; *National Eye Institute* – George McKie (Program Officer); *Oregon Health and Science University, Portland, Oregon, USA* – Kenia Chavez, Travis Redd, Winston Chamberlain; *Pacific Vision Institute of Hawaii, Honolulu, Hawaii, USA* – Angel Cheng, Vivien Tham; *Phramongkutklao Hospital, Bangkok, Thailand –* Wiwan Sansanayudh; *Programme National de Santé Oculaire, Niamey, Niger* – Abba Kaka Hajia Yakoura, Abdou Amza, Abdoul Salam Youssoufou Souley, Adam Nouhou Diori, Beido Nassirou, Boubacar Kadri, Boubacar Mariama, Cissé Mamadou Ibrahim, Lamyne Aboubacar Roufaye, Ramatou Boulhassane, Saley Ali, Zakou Abdou; *Rabin Medical Center, Petah Tikva, Israel* – Lee Goren, Irit Bahar, Ruti Sella; *Sinai Hospital, Baltimore, Maryland, USA* – Clare Kelliher, Laura Green; *Singapore Eye Research Institute, Singapore* – Hon Shing Ong, Jod Mehta, Yu-Chi Liu; *Stanford University School of Medicine* – Benjamin A. Pinsky; *Taipei Veterans General Hospital, Taipei, Taiwan* – De-Kuang Hwang, Nai-Wen Fan; *The University of Sydney, Save Sight Institute, Sydney, Australia* – Hong Sheng Chiong, Javier Lacorzana, Maria Cabrera-Aguas, Stephanie Watson; *University of California Los Angeles Stein Eye Institute, Los Angeles, California, USA* – Edmund Tsui, Nina M. Cherian, Rachel Feit-Leichman, Reginald E. Hughes Jr, Tania Onclinx, Joseph K Privratsky; *University of California San Diego Shiley Eye Institute; La Jolla, California, USA* – Carol Yu, Esmeralda McClean, Iliana Molina; *University of California San Francisco Francis I. Proctor Foundation, San Francisco, California, USA* – Armin Hinterwirth, Cindi Chen, Danny Yu, David Liu, Elodie Lebas, Emily Colby, Gerami Seitzman, Kevin Ruder, Lina Zhong, Michael Deiner, Thomas Abraham, Thomas Lietman, Thuy Doan (Principal Investigator), Travis Porco, Stephen McLeod; *University of California Berkeley School of Optometry, Berkeley, California, USA* – Kuniyoshi Kanai, Meredith Whiteside; *University of Nebraska Medical Center Truhlsen Eye Institute, Omaha, Nebraska, USA* – Steven Yeh, Tolulope Fashina; *University of New Mexico, Albuquerque, New Mexico* – James Chodosh; *University of Papua New Guinea School of Medicine and Health Sciences, Port Moresby, Papua New Guinea* – Bridgit Tarkap, Jambi N. Garap, Magdalene Mangot; *Vanuatu Eye Program, Ministry of Health, Vanuatu* – Edwin Amel, Fasihah Taleo, Johnson Kasso, Kalbule Willie, Madopule Nanu, Prudence Rymill; *World Health Organization* – Anthony W. Solomon
